# The mechanical characterization of the legs, fangs, and prosoma in the spider *Harpactira curvipes* (Pocock 1897)

**DOI:** 10.1038/s41598-022-16307-y

**Published:** 2022-07-29

**Authors:** Sara Residori, Gabriele Greco, Nicola M. Pugno

**Affiliations:** 1 Laboratory for Bioinspired, Bionic, Nano, Meta Materials & Mechanics, Department of Civil, Environmental and Mechanical Engineering, University of Trento, Via Mesiano, 77, 38123 Trento, Italy; 2 School of Engineering and Material Science, Queen Mary University of London, Mile End Road, London, E1 4NS UK

**Keywords:** Polymers, Mechanical properties, Characterization and analytical techniques, Mechanical engineering

## Abstract

The exoskeleton of spiders is the primary structure that interacts with the external mechanical stimuli, thus playing a crucial role in spider life. In particular, fangs, legs, and prosoma are the main rigid structures of the exoskeleton and their properties must be measured to better understand their mechanical behaviours. Here we investigate, by means of nanoindentation, the mechanical properties of the external sclerotized cuticles of such parts in the spider *Harpactira curvipes*. Interestingly, the results show that the leg’s cuticle is stiffer than the prosoma and has a stiffness similar to the one of the tip fangs. This could be explained by the legs’ function in perceiving vibrations that could be facilitated by higher stiffness. From a broader perspective, this characterization could help to understand how the same basic material (the cuticle, i.e. mainly composed of chitin) can be tuned to achieve different mechanical functions, which improves the animal’s adaptation to specific evolutive requirements. We, thus, hope that this work stimulates further comparative analysis. Moreover, these results may also be potentially important to inspire the design of graded materials with superior mechanical properties.

## Introduction

The interaction among animals and their habitats is important for all living organisms, spiders included. In this sense, the arthropods’ cuticle not only provides protection, attachment sites for muscles, joints, and a transmitter of muscles forces onto the substrates the animals move on, but it also provides a platform for the sensors that perceive the surrounding^1,2^. These mainly work with chemical and mechanical stimuli^3^ that can be detected by specific structures, such as lyriform organs^4^ or seate^5^, found on the surface of the exoskeleton^6^.

The exoskeleton is a composite made of rigid chitin nanofibrils embedded in a protein matrix^7^. In nature, a single composite material could have different mechanical properties and functions that may depend on its eventual hierarchical structure^8^. Moreover, studying the mechanical properties of functional anatomical parts is important for ecology, which benefits from the intersection with biomechanics that provides insights to improve the knowledge on behavioural ecology^9^.

As an example, spider fangs have recently been a research object for their crucial functions in spider life^10^. The fangs present a well-defined architecture composed of a structural/material gradient that is adapted to allow the spider to feed, defend itself and dig, all of which require resistance to wear^11^. Interestingly, the optimization of such functions has been recently linked to the metal inclusions in the animals’ cuticles^12^.

Politi et al.^10^ explored the internal lamella of the spider's fangs (whose layers ranging from 100 µm to 30 nm in thickness) and their mechanical properties with a Scanning Acoustic Microscope (SAM) and nanoindentation (the hardness was about 0.6 GPa and Young’s modulus of about 10 GPa). The comparison between measurements performed on longitudinal and transversal fang cross-sections indicated that fibre orientation contributes in determining the mechanical properties. The mechanics of spiders' fangs were also investigated through computed tomography, analytical models, and numerical simulations with finite elements^13^. Erko et al.^14^ analysed the nano composition of the structural hierarchy and its metal reinforcing elements by comparing the mechanical properties (nanoindentation) and the structural organization (X-rays) of the cuticle of the wandering spider *Cupiennius salei*. The experimental results revealed that the tip of the spider fang, which is used to inject venom into the prey, is harder and stiffer with respect to the basal.

Aside from the fangs, there could be other exoskeleton regions of interest, the cuticle being an example of a reinforced microfibre material^14,15^. Furthermore, the carapace and legs are stiff regions that could be as crucial as fangs in spider’s life, e.g. to detect vibrations or to transmit forces to the ground^16–18^. Until now, mainly the spider *C. salei* has been studied. Moreover, the elements of the spider’s body (fangs^10^, claws^19^, legs^17,18^) have been mechanically analysed individually from different specimens. Thus, no previous study compared the different mechanical properties of several parts of the exoskeleton tested from the same individual. In this context, a comparison of the mechanical properties of different body parts may give some insights to better understand their functions, as it has been suggested for insects^20^.

Here, we present the comparison of the mechanical properties of different parts for the species *Harpactira curvipes* (Pocock 1897^21^), which is not closely related to *Cupiennius salei*. The exoskeleton parts that have been selected were the ones most exposed to continuous external stimuli: legs, prosoma/carapace, and fangs^22^. Through nanoindentation, we provide a map of their mechanical properties, which could be used as insight for a better understanding of spider biomechanics, e.g. locomotion and sensing^2,3,23^. Finally, this study may contribute to the design of bio-inspired materials with superior and tuneable mechanical performances^24–28^.

## Methods and materials

### Spider

The spiders under study were the Theraphosidae *Harpactira curvipes* (Pocock, 1897^21^), which live mainly in South Africa^21^*.* The animals were two females of about 70 g, raised and born in captivity from a local breeder, and were kept in the laboratory in terrarium. The environmental conditions were controlled to have inside the terrarium, 25–27 °C during the day and 22–23 °C during the night. The humidity of 70% was achieved by watering weekly the spider. They were fed weekly with cockroaches and crickets, raised and bred in the laboratory. The analysed parts of the body were dissected from the dead animal (euthanized in ethanol), preserved in ethanol (70% w/v), and kept in the refrigerator (4–8 °C). In particular, the selected cuticle’s body parts were: both fangs (both of them), metatarsus of the I pair of legs, and the spider’s prosoma.

### Specimen preparation

The samples’ preparation followed common protocols for the nanoindentation of biological materials, spiders tissue included^10,12–14,29–33^. The dissected parts were positioned in a plexiglass sample holder and included in an epoxy resin matrix, which was purchased from Hardrock 554® (Remet). The resin was let solidify at room temperature for about 23 °C for two days. To expose the cross section of the region of interest, the samples were then polished mechanically with the support of a Remet LS2 by following the procedure described in^34^ (minimum roughness achievable of about 5 nm). The polish procedure ensures the flatness of the surface, eliminating the roughness that could affect the measurements. For the leg and the prosoma parts, we indented the external sclerotized layer of the cuticle.

### Nanoindentation tests

The samples were then mounted into an iNano®Nanoindenter (Nanomechanics Inc.) equipped with a Berkovich tip^35^. The declared sensitivity of the machine is $3$ nN for the load and $0.1$ nm for the displacement. The data set was obtained through indentations performed with two methods: single and mapping test, each for a maximum of 45 mN loads. The used mapping method (Nanoblitz 3d, Nanomechanics Inc.) involved a 200 μm × 200 μm square with 10 × 10 equidistant indentation points inside. On the basal longitudinal section of the fang and on the resin used to prepare the nanoindentation samples, more tests were performed with different spacing (10, 30, 20, and 40 μm). This was done to check the effect of the distance between indentation points on the mechanical properties. The values of the mechanical properties were obtained according to the theory of Oliver-Pharr^36^. In brief, the hardness was evaluated with the formula

$$H=\frac{P}{A}$$

where *P* was the maximum load of 45 mN and was the projected contact area (in this case evaluated for a Berkovich tip). The modulus was evaluated as:

$$E=\frac{1}{2}\frac{dP}{dh}\frac{\sqrt{\pi }}{\sqrt{A}}$$

where $dP/dh$ was the slope of the unloading section of the elastic–plastic load curve as a function of the indentation depth. Since Poisson’s coefficient ranges between 0.2 and 0.49 for chitin-based materials^37^, we considered 0.33 as a representative value for this hard material as suggested by Fischer-Cripps^34^. All the tests were performed in a room with controlled environmental parameters (19–21 °C, 15–30% RH).

### Optical images

The optical images were obtained with an optical microscope (Olympus BX61/62TRF) Olympus Steam Image Analysis software.

### Scanning electron microscopy

To check the validity of the nanoindentations results (i.e. control of the print), a Scanning Electron Microscope (SEM) investigation was carried out. Samples were observed with a Field Emission SEM (FE-SEM, Supra 40/40VP, Zeiss, Germany) after coating with Pt/Pd (80:20) in a reduced argon atmosphere by means of a Quora Q150 machine.

### Atomic force microscopy

To check the validity of the nanoindentations results (i.e. control of the print and roughness), an Atomic Force Microscope (AFM) investigation was carried out. The AFM used to obtain the images is a NT-MDT Smena scanner. The environmental conditions at which we operated were controlled and with a temperature of 24 °C and relative humidity of 71%. To obtain topography we used, in semi-contact mode, a NT-MDT NSG-11B tip (10 nm nominal tip radius, resonance frequency of 181 kHz, and force constant between 2.5 and 10 N/m). AFM data were analyzed with the support of Gwyddion and IA_P9 free software^38^. The values of the surface’s roughness were computed both for the average (Ra) and mean root square (Rq) value, following British standard ISO 4287:2000.

### Statistical analysis

Results are reported as mean ± standard deviation. Collected data were analysed through the analysis of variance (pairwise comparison of the values of Young’s modulus and hardness). The statistical test was the one-way ANOVA test and 95% confidence limits were assessed, referring to a two-tailed Gaussian distribution. These parameters were used to verify the null hypothesis, i.e. all the data set come from the same distribution and have the same mean value. The sum of residual squares (*SS*) was evaluated as:

$${SS}_{1}=\sum_{i=1}^{r}c {(\overline{{x }_{i}}-\overline{x })}^{2}$$

$${SS}_{2}=\sum_{i=1}^{r}\sum_{j=1}^{c}{({x}_{ij}-\overline{{x }_{i}})}^{2}$$

where *r* is the number of different samples under consideration, *c* is the number of tests of the same sample, $\overline{x }$ is the mean value of all data, $\overline{{x }_{i}}$ is the mean value within the group, and *x* is the single value. The ratio of variances was calculated as:

$$F=\frac{\frac{{SS}_{1}}{r-1}}{\frac{{SS}_{2}}{c-r}}$$

This ratio followed a Fisher distribution with a significance level of 5%. If *F* > ${F}_{cr}$, the null hypothesis was rejected and the difference among the data set was considered significant. The p-value was computed in Excel®, and the difference was considered significant if it was lower than 0.05.

### Effect size

Larger samples are more likely to give statistically significant differences under the ANOVA test^39^. In order to estimate a measure of the magnitude of such difference, we calculated the Effect Size (ES). We based our analysis on the parameters introduced by Cohen^40^. By assuming that the two compared populations have the same variance, a pooled standard deviation can be defined as:

$$s=\sqrt{\frac{\left({n}_{1}-1\right){s}_{1}^{2}+\left({n}_{2}-1\right){s}_{2}^{2}}{{n}_{1}+{n}_{2}-2}}$$

where *n*_*1*_ and *n*_*2*_ are the dimensions of the two compared groups, and *s*_*1*_ and *s*_*2*_ are their standard deviations. Cohen defined the parameter *d*_*c*_ as a function of *s* according to:

$${d}_{c}=\frac{{m}_{1}-{m}_{2}}{s}$$

where *m*_1_ and *m*_2_ are the means of the two groups, respectively. As defined by Cohen and Sawiloski^40^, the parameter *d*_*c*_ qualitatively describes the magnitude of the difference of the means as very small (0.01 ≤ *d*_*c*_ < 0.20, i.e., circa 100% distributions’ overlap), small (0.20 ≤ *d*_*c*_ < 0.50, i.e., circa 85% distributions’ overlap), medium (0.50 ≤ *d*_*c*_ < 0.80, i.e., circa 67% distributions’ overlap), large (0.8 ≤ *d*_*c*_ < 1.20, i.e., circa 53% distributions’ overlap), very large (1.20 ≤ *d*_*c*_ < 2.00, i.e., circa 40% distributions’ overlap), and huge (*d*_*c*_ ≥ 2.00, i.e., circa 19% distributions’ overlap).

## Results

**Figure 1:**
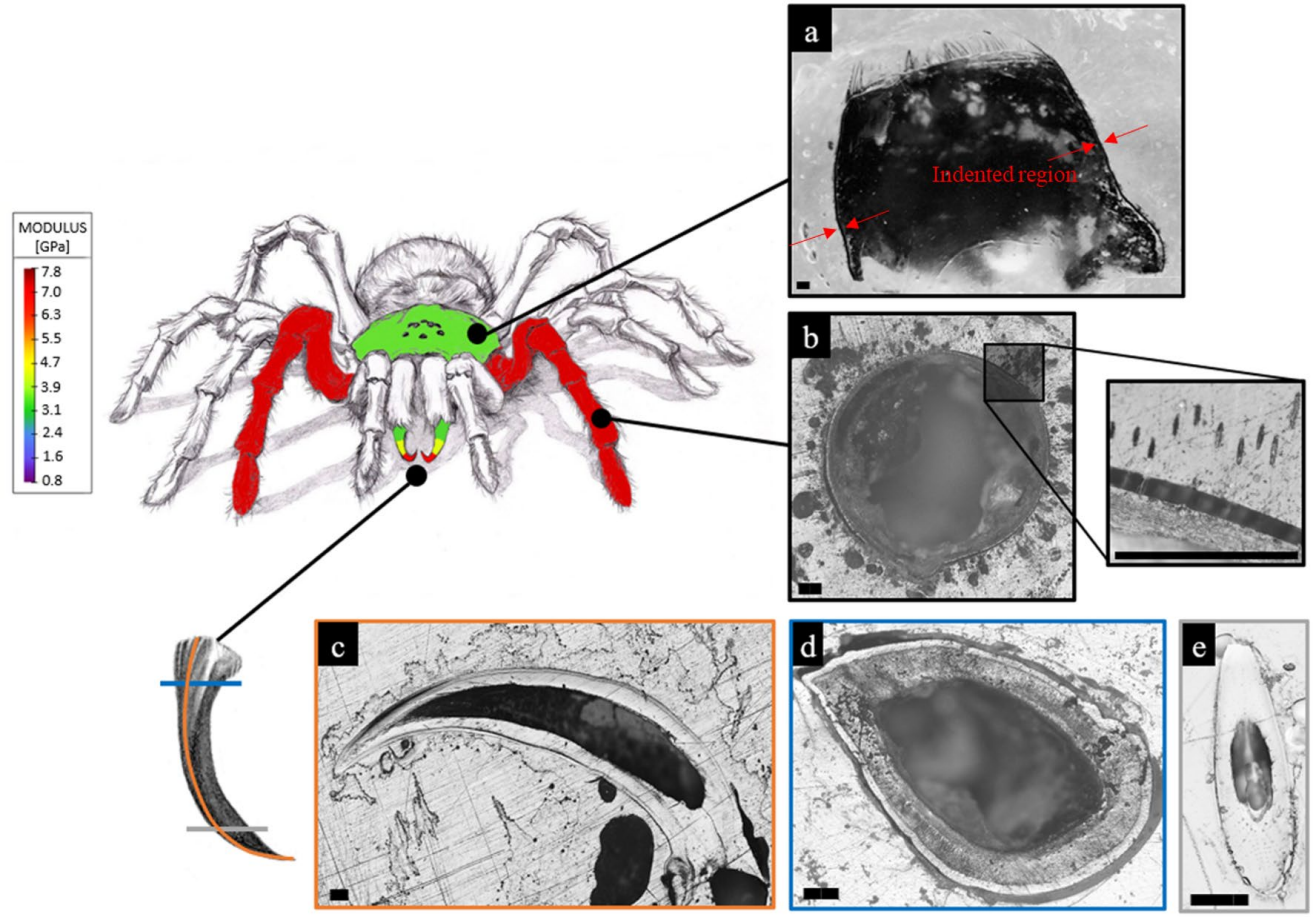
Representative drawing of the spider with the sections of interest. Coloured areas represent different results of the Young’s modulus measured by nanoindentation. (**a**) Section of the prosoma in the longitudinal direction (here the indented region is indicated). (**b**) Section of the leg in the transversal section with a detail of the hair section. (**c**) Section of the fang in the longitudinal direction. (**d**) Section of the fang in the transversal direction, on the base. (**e**) Section of the fang in the transversal direction, on the tip. The colour filling of the spider schematic is to be considered illustrative, as the exoskeleton is composed of many other parts, e.g. hairs, joints. Scale bars: 200 µm.

The spider exoskeleton was analysed in different parts and the mapping of mechanical properties (i.e. Young’s modulus and hardness) was obtained through single and multipoint nanoindentations (Fig. 1). Although our nanoindentation setup makes it impossible to test fresh tissue, since it requires their inclusion in resin to mechanically stabilize the sample and a polish procedure to ensure the maximal flatness of the surface, it is similar to previous reported protocols^10,14,29–33^. Since the proximity of two indentations point affects the mechanical properties^34^, different spacing have been tested for both the basal longitudinal section of the fang and the resin used to prepare the samples. The results are depicted in Table S1, where no significant differences occurred between the values 10, 30, and 40 μm and those at 20 μm, which have been used for further analysis.

**Figure 2:**
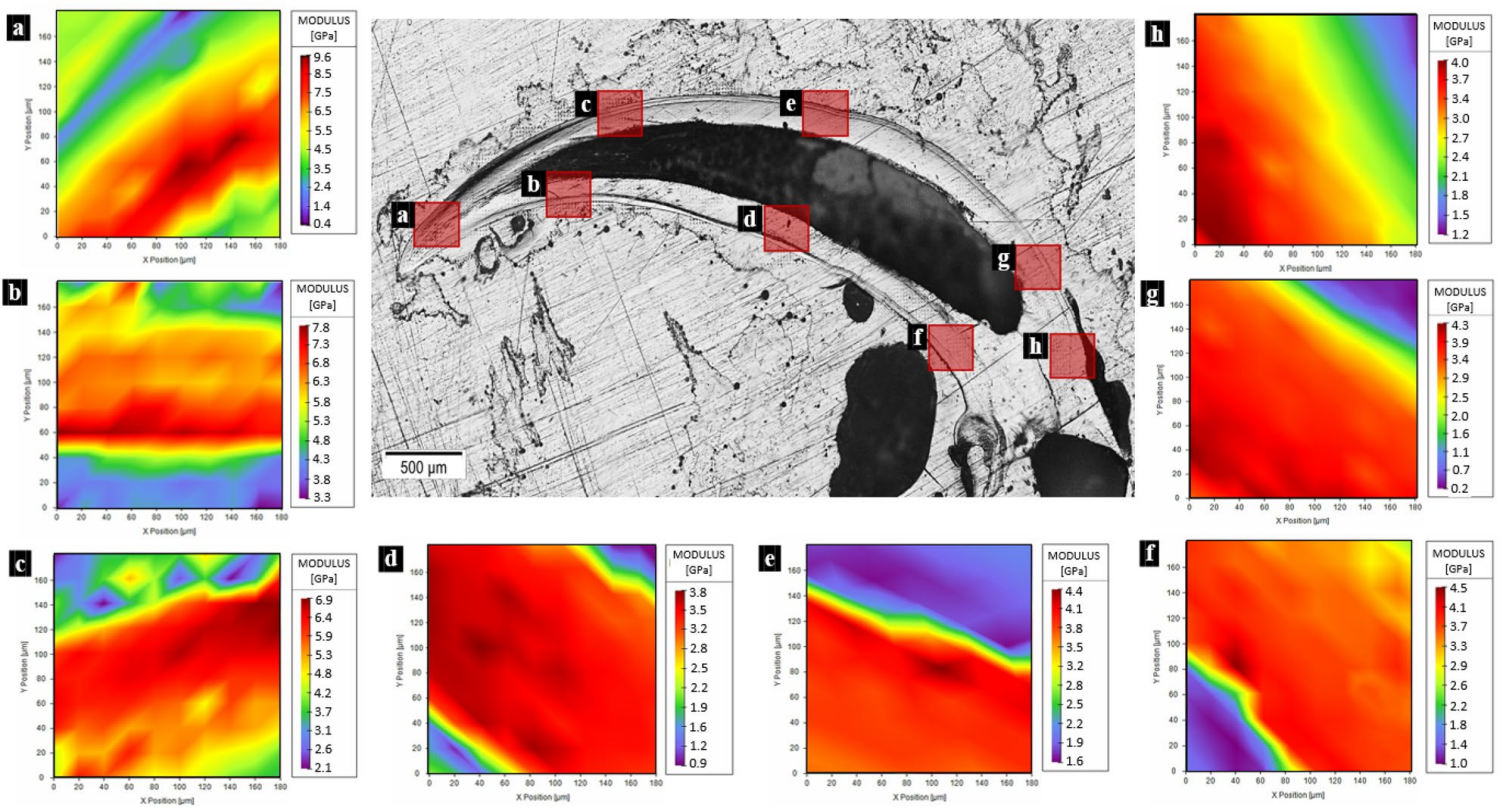
Map of the Young’s modulus in different sections of the spider fang in the longitudinal direction. In general, the outer layer of the sclerotized cuticle is stiffer than the internal. Images generated with the support of Nanoblitz 3d, Nanomechanics Inc.

**Figure 3:**
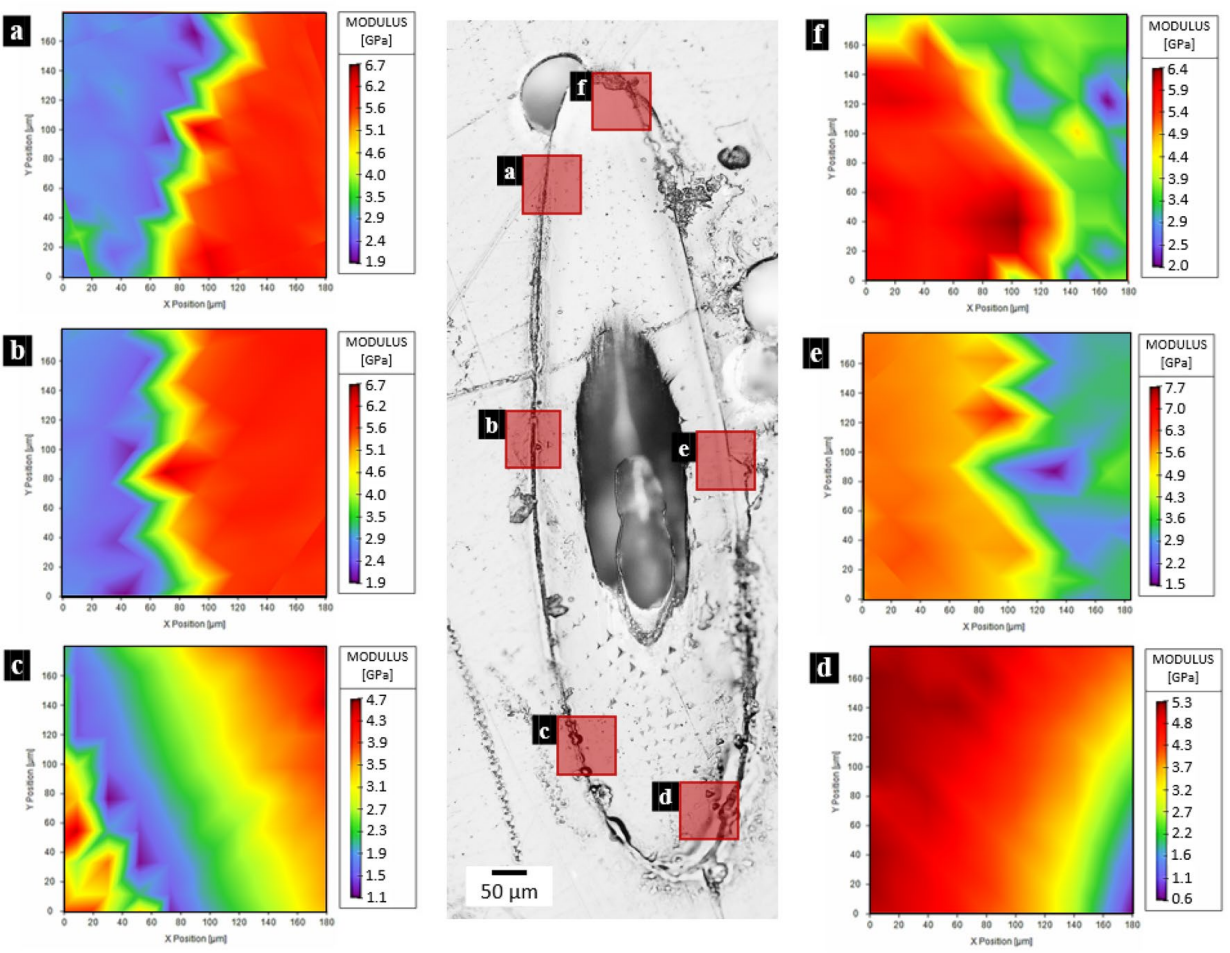
Map of Young’s modulus of the spider fang in the transversal section on the tip. In general, the outer layer of the sclerotized cuticle is stiffer than the internal. Images generated with the support of Nanoblitz 3d, Nanomechanics Inc.

**Figure 4:**
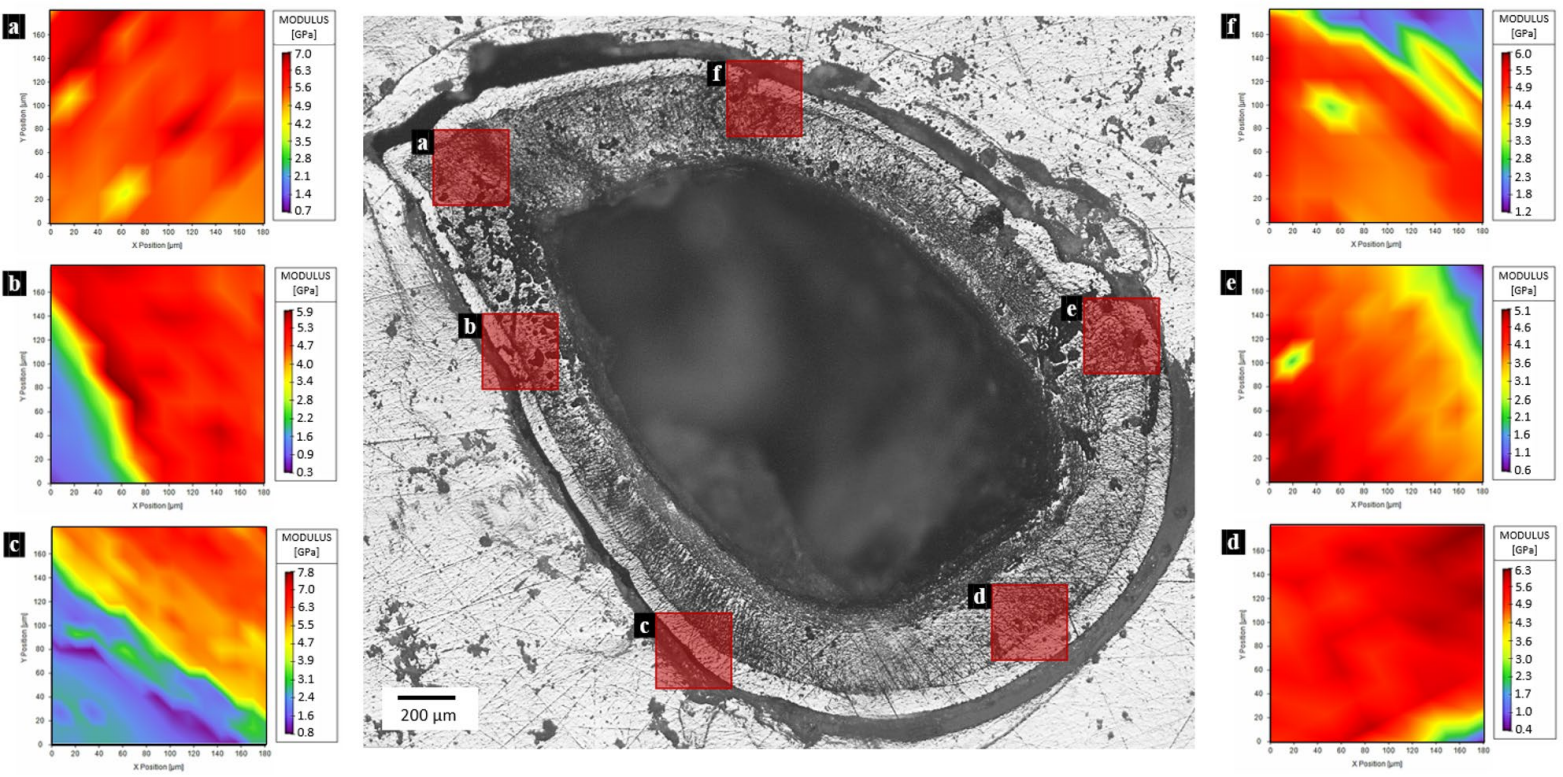
Map of the Young’s modulus of the spider fang in the transversal section on the base. Images generated with the support of Nanoblitz 3d, Nanomechanics Inc.

**Figure 5:**
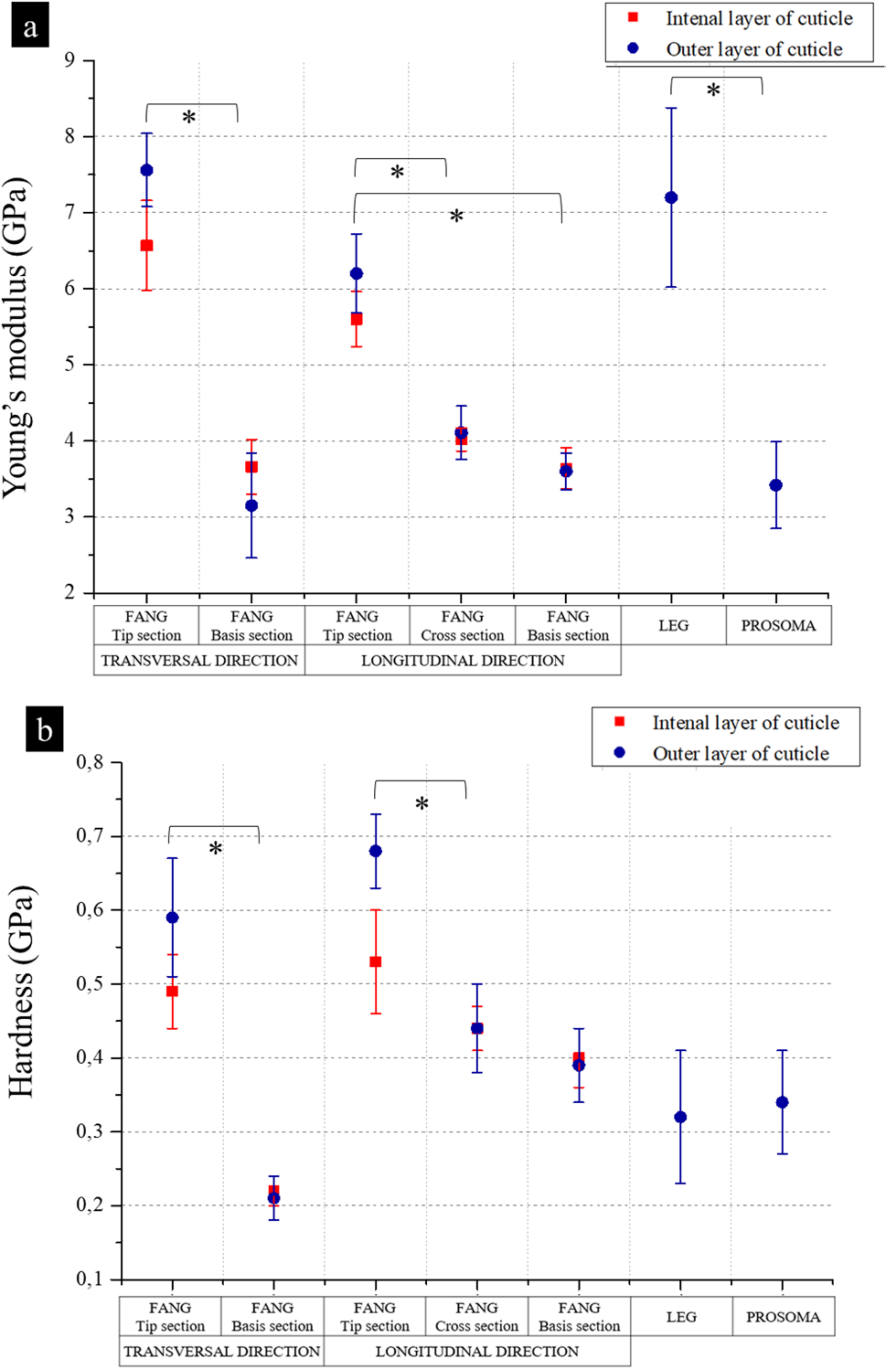
Values of the Young’s modulus (**a**) and hardness (**b**) in each analysed section of the spider. Stars indicate significant differences (p-value < 0.5) and are valid for both external and internal cuticle layers.

The mechanical properties of the prosoma (Fig. 1a) and legs (Fig. 1b) sclerotized cuticle are listed in Table S2-S3, and they were obtained by single indentation points. On the other hand, in the fangs’ cuticle, the investigation was performed in different directions (Fig. 1c–e): the longitudinal and the transversal. In these, two regions (based on Fig. 1d) can be distinguished: the brighter is the outer layers of fang’s cuticle (OL) and the darker is the internal layers of fang’s cuticle (IL). The results regarding the tip section on the longitudinal direction are depicted in Fig. 2a–c, Table S4, and Figure S1. The mechanical properties of the fang in the same direction, but in the middle region are depicted in Fig. 2d,e, Table S4, and Figure S1. The mechanical properties of the section at the base of the fang in the longitudinal direction are presented in Fig. 2f–h, Table S4, and Figure S1. For the fang tip, the results are depicted in Fig. 3, Table S5, and Figure S2 and for the basal section, Fig. 4, Table S5, and Figure S3 show the values of the measured mechanical properties. The comparison of the mechanical properties of the different body parts was performed through ANOVA and ES tests (Tables S6 and S7) (Fig. 5). In general, the average values of hardness and Young’s modulus of the OL were significantly higher than those of the IL at the tip sections. Moreover, for both the transversal and the longitudinal direction, the tip section has significantly higher Young’s modulus and hardness than the basal. The Young’s modulus of the legs’ cuticle was significantly higher than the cuticle of the prosoma. Surprisingly, the legs displayed a similar value of Young’s modulus with respect to the tip section of the fangs’ cuticle and were significantly stiffer than their basal sections. On the other hand, the tip section of the fangs’ cuticle was significantly harder than the leg one. Moreover, within the fang, the hardness and Young’s modulus in OL were overall higher than those in IL, especially at the tip.

**Figure 6:**
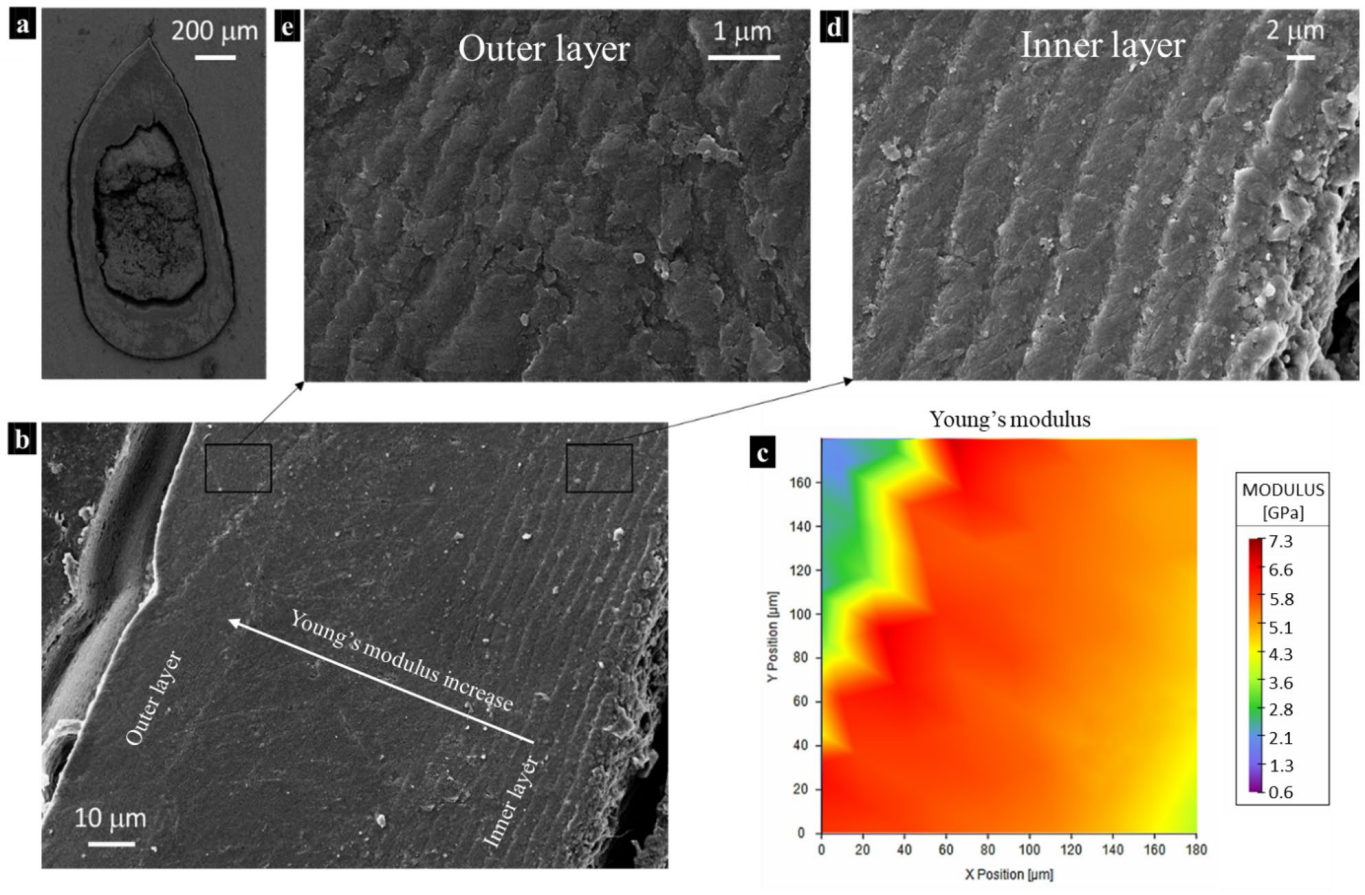
(**a**) Transversal section of the spider fang at its basis by SEM. (**b**) SEM image of the lateral edge of the fang. (**c**) The Young’s modulus map of the region depicted in (**b**) by nanoindentation technique. (**d**) SEM image of the inner cuticle layers of the fang. (**e**) SEM image of the outer cuticle layer of the fang. Images generated with the support of Nanoblitz 3d, Nanomechanics Inc.

Through the SEM and AFM analysis, the quality of the surface was evaluated. The values of the average roughness (R_a_) and mean root square roughness (R_q_) obtained with AFM were 6.7 ± 0.9 nm and 8.5 ± 1.1 nm (Table S8) respectively, which are lower concerning the nanoindentation depths (Tables S2-S5). AFM and SEM techniques were also used to check the residual impression of the indenter (Figure S4). In particular, it has been observed the occurrence of plasticity and the absence of cracks’, which can be due to surrounding structural failure that affects the mechanical properties^34^. Furthermore, the SEM investigation revealed insights into the micro-structure of the spider’s fang. In accordance with the literature^15^, a diversification of the stacked layers was observed. In particular, going from the inside to the outside of the section, an overlap of the internal layers of constant thickness was present. Afterward, there was a homogeneous layer with parallel structures and finally the outer cuticle layer. Interestingly, the reduced spacing among these layers seems to coincide with an increase in Young’s modulus and hardness (Fig. 6). Finally, we noticed that different elements presented various microstructural organizations. The more structured elements, i.e. fangs, revealed a clear distinction between the inner and the outer cuticle’s layer (Fig. 6). In particular, the IL constituted the most extended area of the fang, with a different organization of layers of chitin (Fig. 6d). On the other hand, the OL was composed of compact chitin stacking with a lower thickness (Fig. 6e). In the other considered elements, this distinction was not visible, and the mechanical properties were not different along the section.

## Discussion

The nanoindentation technique allows researchers to perform local maps of mechanical properties, which are important to understand the mechanics of artificial and natural structures^29–33,35,41,42^. In this work, the investigation was focused on different elements of the spider’s exoskeleton for which we expect different mechanical properties: legs, prosoma, and fangs. The reason of this can be ascribed to the different cuticular structures present in these parts. Indeed, the spider cuticle is similar to the insects one that consists in various layers (exo-, meso-, and endocuticle)^1,43^. These are present in different proportions depending on the considered body part of the spider^15^, and have various level of sclerotization (i.e. mesocuticle is more sclerotized than endocuticle) and microstructural organization, two factors that both define the mechanical properties of the exoskeleton^44–46^.

In general, the reported results are aligned with previous indentation studies on spider fang’s tissue^10–14^. However, no previous work reports the comparison of mechanical properties of different body parts. This work aims to fill this gap, providing a comparison of the mechanical properties of the sclerotized layer of cuticle in the legs, prosoma, and fangs.

The prosoma exoskeleton has the main functions to shield some vital organs as well as protect the spider from quick dehydration^47^. Moreover, it is also the tagma responsible for locomotion, feeding, and sensing, since the prosoma’s cuticle is the base for the animal’s haemolymph pressure pump (i.e. the drive for the extension of two major joints of all of their legs and pedipalps)^1,3^. Legs are crucial for locomotion^3^ and sensing purposes since they host the major part of mechanical and chemical sensors^4^. On the other hand, fangs are indispensable to feed, dig, and defend the spider^10^. Thus, the fangs are the ones exposed to major external stresses, which require a particular chitin structure, consisting in a lamellar architecture of the internal cuticle’s layers, confirmed by the here reported SEM images^10^. This structural design results in mechanical properties that​ depend on the thickness and orientation of lamellas^13^. This was confirmed here through nanoindentation, which showed a gradient of Young’s modulus and hardness. In particular, the closer the cuticle’s layers the higher Young’s modulus and hardness were measured. Moreover, as already demonstrated in other studies for other species^10,13^, the fang’s tip presented higher values of the hardness and Young’s modulus with respect to the base. This is related to the biological function of the tip, i.e. the penetration of surfaces as well as the durability and wear resistance of the tool^19^. From this point of view, a recent work showed that the actions of penetrating surfaces as well as resisting wear and tear are aided by metals inclusion in the cuticle^12^. This is in accordance with a previous study, which showed the presence of metal ions in the fangs of the spiders that act as reinforcing elements^12,14^. Moreover, the different microstructural organization of the cuticle body parts (legs, prosoma, outer layer, and inner layer of the fangs) has been related to the polymorphic forms differentiated into their crystal structure^48^. Interestingly, the different microstructural organization of the cuticle as well as the gradient of the mechanical properties is found also in beetles^49^, highlighting the common evolutionary strategies (i.e. microstructural organization) to optimize the biomechanical performances of anatomical parts.

The OL of the fangs was stiffer and harder than IL which is in agreement with the prediction of previous studies^13^. Interestingly, the metatarsus of the I pair legs’ cuticle was found as stiff as the fangs’ one. The legs of the Theraphosidae host the majority of lyriform organs^47^, which are sensory systems with the aim to detect vibrations^4^. These, in different shapes and numbers up to the considered species, deform because of the detected vibration and transmit the relative signal by means of the nerves. The high stiffness of the legs’ cuticle, found in this work, may be thus important to facilitate the transmission of mechanical stimuli^50,51^, which may depend on both temperature and humidity^17^, to slit sensilla. Indeed, spider lyriform organs have high mechanical sensitivity, which also depends on their exponential stiffening behaviour, i.e. the relation between the cuticle’s stress and strain follows an exponential curve. This allows the animals to detect a wide range of vibration amplitudes over four orders of magnitude in frequency as low as 0.1 Hz and as high as several kHz^15–18^, which is what commonly is present in nature^18^. For these reasons, the stiffer leg cuticle in *Harpactira curvipes* may aid the perception of vibrations.

Moreover, we do not exclude that stiff cuticle tissue is beneficial for the spiders’ locomotion. This requires high differences in the pressure applied by haemolymph, which flows inside the legs, on the cuticle^3^. In this context, a recent work^23^ shows that spider haemolymph behaves as a shear-thinning non-Newtonian fluid, whose fluid behaviour index is 0.5 (usually < 1 for pseudoplastic fluids, = 1 for Newtonian fluids, and > 1 for dilatant fluids). This means that the higher the shear stress applied on the haemolymph, the lower is its viscosity. During spiders’ legs fast movements, there is a quick increase of pressure in the haemolymph, which results in higher shear stress, induced by the interactions with the cuticles’ walls^3^. In this way, the stiffer the walls are, the higher is the shear stress on the haemolymph^52^, which results to be less viscous, flows better in the joints, and facilitates locomotion. Moreover, a more rigid cuticle sustains better the continuous changes in pressure within the legs^23,28^. These results may be helpful to design bio-inspired hard or hard/soft systems, such as cutting tools or soft actuators, providing an example of how an external rigid layer may help in the development even of soft actuators. In particular, the optimized alternation between soft and rigid tissue can improve actuation mechanisms (e.g. hydraulic) aiding the speed, the force output, the displacement, and the efficiency of artificial joints by localizing the pressure of the actuating fluid preserving hardness^28^.

Although it is very challenging to have a mechanical characterization of all spiders’ body parts (including microstructures such as hairs, slit sensilla, joints, and so on), this study provides an example of the importance in performing such comparative analysis, since it confirms previous studies’ results and offers a starting point for future discussion.

### The role of hydration in affecting nanoindentation results

Nanoindentation experiments were not performed on fresh samples, which were here dissected from animals preserved in ethanol (70%) and kept in the refrigerator (4–8 °C). The hydration status strongly affects the mechanical properties of arthropods cuticle^53,54^, as deeply investigated for insects in Klocke et al.^55^. In that study, it is reported a significant reduction of hardness and Young’s modulus (up to a factor of 9 and 7.4 respectively) in the hydrated cuticle with respect to the dry one. Moreover, it is highlighted that in some cases the relative differences between meso/endo cuticle can be reverted if the samples were dried.

To investigate hydrated samples by means of nanoindentation it is mandatory to use specific instruments in which experiments in fluid are allowed. Indeed, the state of hydration must be maintained during the test, and this is not possible with most of the available devices^34^. Nonetheless, there are instruments and techniques that give the possibility to test samples while fully submerged in fluid^56^. However, these techniques should also be coupled with a method that takes into account the capillary forces that are known to interfere with sample surface detection^57^ and that strongly depend on the indenter geometry^58^. For this reason, to perform such tests, special indenter probes are used for fluid immersed-samples^59^. Finally, a wet sample may display viscoelastic unloading response when the probe is withdrawn^60^. In this sense, the well-known and established method developed by Oliver and Pharr^36^, designed for dry samples, is based on the assumption that the unloading of the sample is elastic. Thus, the calculation of the mechanical properties of hydrated samples should be done also considering their viscoelasticity.

For the sake of clarity, it is important to discuss what are the possible effects of hydration on the results of this work. Klocke et al.^55^ found that endo/meso cuticle are those mostly affected by water, whereas exocuticle is less affected. Following Barth^15^ and Foelix^1^ and looking at the indentation regions of this study, it is possible to say that mostly meso/endo cuticle were here transversally tested^55^. In this context, if we assume that water affects the mechanical properties at the same level as described in Klocke et al.^55^, for both the Young’s modulus and the hardness we expect that the difference between dry and wet samples will not be enough to change the trends observed in this work. Indeed, the relative differences between the meso/endo cuticle are 30% and 10% in the dry state for Young’s modulus and hardness respectively, whereas in the wet state such differences are 17% and 100%. These relative differences are not comparable to the ones that we observed among the different regions of the fang (e.g. basal and tip differ for a factor ~ 3, Fig. 5). On the other hand, this cannot be said for the differences between the external and internal layers of the cuticle. Nonetheless, this does not affect the finding of the remarkable stiffness of the metatarsus cuticle, where no gradient was found (Fig. 5).

In finding the best technique to store cuticle samples, Aberle et al.^61^ suggested that freezing them preserved better their mechanical properties, which is in common with octopuses flesh^62^ and that has been also recently used for locusts^54^. On the other hand, they also show that the moduli of the sample preserved in ethanol are lower (up to three times) with respect to the fresh ones. Before being embedded in resin, the samples tested in this work were stored in ethanol, which may have balanced the dehydration effect described earlier^55^.

When we designed the experiments for this work, the published studies related to spiders were considered. In Tadayon et al.^19^ the species *C. salei* was analysed, testing the samples in both dry and wet conditions. This was done using an instrument designed for the purpose that implemented Oliver and Pharr^36^ method. In that case, the dry samples reported an increase of Young’s modulus and hardness compared to the hydrated ones of about 41% and 35% respectively. These values have been used to estimate the mechanical properties in hydrated state in this study (Figure S5). Nonetheless, they show that the gradients inside the fangs were preserved, which further supports the validity of our study. This is also supported by the studies conducted by Erko et al.^14^ and Politi et al.^10^, in which *C. salei* tissue were preserved in Ethanol 70% and stored at 4–8 °C, and then tested in dry conditions with a Berkovich tip and using Oliver and Pharr^36^ method, as it has been done in this study.

Thus, the mechanical properties here presented are to be considered as comparative with respect to the literature, and differences with respect to fresh tissue should be expected. We hope that this work will stimulate further discussion on the delicate topic of measuring the mechanical properties of arthropods cuticle, as thoroughly dealt in Stamm et al.^20^.

## Conclusion

In this study, we provide a map of the mechanical properties by means of nanoindentation of different sclerotized cuticular elements of the spider body. This helps to understand the intra-individual differences of the mechanical functions and properties of the cuticular parts under study (namely legs, prosoma, and fangs). We confirmed the results of previous studies on a different species and we showed that spider legs’ cuticle is stiffer than prosoma and as stiff as the fang tips. This research offers new data on spider’s crucial biomechanical structures, which are essential to understand spider biomechanics (e.g. locomotion or sensing), and it offers a case study on which build further discussion and possible design of new bio-inspired structures.

## Supplementary Information

Supplementary Information 1.

Supplementary Information 2.

Supplementary Information 3.

## Data Availability

All data generated or analyzed during this study are included in this published article and its supplementary information files.

## References

[1] Foelix, R. *Biology of Spider*. *Oxford University Press* vol. 53 (2011).

[2] Weihmann T, Goetzke HH, Günther M (2016). Requirements and limits of anatomy-based predictions of locomotion in terrestrial arthropods with emphasis on arachnids. J. Paleontol..

[3] Nentwig W (2013). Spider ecophysiology. Spider Ecophysiol..

[4] Barth. *Spiders world: senses and behaviour*. vol. 133 (2002).

[5] Guarino R, Greco G, Mazzolai B, Pugno NM (2019). Fluid-structure interaction study of spider’ s hair flow-sensing system. Mater. Today Proc..

[6] Fabritius H (2011). Chitin in the exoskeletons of arthropoda: from ancient design to novel materials. Science.

[7] Khor E (2014). Chitin and chitosan biomaterials. Chitin.

[8] Wegst UGK, Bai H, Saiz E, Tomsia AP, Ritchie RO (2015). Bioinspired structural materials. Nat. Mater..

[9] Bauer U, Poppinga S, Müller UK (2020). Mechanical ecology—taking biomechanics to the field. Integr. Comp. Biol..

[10] Politi Y (2012). A spider’s fang: How to design an injection needle using chitin-based composite material. Adv. Funct. Mater..

[11] Moon MJ, Yu MH (2007). Fine structure of the chelicera in the spider Nephila clavata. Entomol. Res..

[12] Schofield RMS (2021). The homogenous alternative to biomineralization: Zn- and Mn-rich materials enable sharp organismal “tools” that reduce force requirements. Sci. Rep..

[13] Bar-On B, Barth FG, Fratzl P, Politi Y (2014). Multiscale structural gradients enhance the biomechanical functionality of the spider fang. Nat. Commun..

[14] Erko M (2013). Structural and mechanical properties of the arthropod cuticle: Comparison between the fang of the spider Cupiennius salei and the carapace of American lobster Homarus americanus. J. Struct. Biol..

[15] Barth FG (1973). Microfiber reinforcement of an arthropod cuticle - Laminated composite material in biology. Zeitschrift für Zellforsch. und Mikroskopische Anat..

[16] Seo JH, Kim KJ, Kim H, Moon MJ (2020). Lyriform vibration receptors in the web—building spider, Nephila clavata (Araneidae: Araneae: Arachnida ). Entomol. Res..

[17] Young SL (2014). A spider ’ s biological vibration filter: Micromechanical characteristics of a biomaterial surface. Acta Biomater..

[18] Schaber CF, Gorb SN, Barth FG (2012). Force transformation in spider strain sensors: white light interferometry. J. R. Soc. Interface.

[19] Tadayon M (2020). Adaptations for wear resistance and damage resilience: Micromechanics of spider cuticular “tools”. Adv. Funct. Mater..

[20] Stamm K, Saltin BD, Dirks JH (2021). Biomechanics of insect cuticle: an interdisciplinary experimental challenge. Appl. Phys. A Mater. Sci. Process..

[21] Pocock RI (1897). On the spiders of the suborder Mygalomorphae from the Ethiopian Region, contained in the collection of the British Museum. Proc. Zool. Soc. London.

[22] Ram J, Michalik P (2019). The spider anatomy ontology (SPD)—A versatile tool to link anatomy with cross-disciplinary data. Diversity.

[23] Gőttler C (2021). Fluid mechanics and rheology of the jumping spider body fluid. Soft Matter.

[24] Kang D (2014). Ultrasensitive mechanical crack-based sensor inspired by the spider sensory system. Nature.

[25] Luo C (2017). Highly sensitive, durable, and multifunctional sensor inspired by a spider. ACS.

[26] Kim T (2018). Polyimide encapsulation of spider-inspired crack-based sensors for durability improvement. Appl. Sci..

[27] Liu Y (2020). Spider-inspired ultra-sensitive flexible vibration sensor for multifunctional sensing. ACS Appl. Mater. Interfaces.

[28] Kellaris N (2021). Spider-inspired electrohydraulic actuators for fast soft-actuated joints. Adv. Sci..

[29] Sachs C, Fabritius H, Raabe D (2006). Hardness and elastic properties of dehydrated cuticle from the lobster Homarus americanus obtained by nanoindentation. J. Mater. Res..

[30] Sun J, Tong J (2007). Fracture toughness properties of three different biomaterials measured by nanoindentation. J. Bionic Eng..

[31] Sun J, Wu W, Ling M, Bhushan B, Tong J (2016). A dynamic nanoindentation technique to investigate the nanomechanical properties of a colored beetle. RSC Adv..

[32] Hassanzadeh P (2014). Mechanical properties of self-assembled chitin nanofiber networks. J. Mater. Chem. B.

[33] Juárez-de la Rosa BA, Muñoz-Saldaña J, Torres-Torres D, Ardisson PL, Alvarado-Gil JJ (2012). Nanoindentation characterization of the micro-lamellar arrangement of black coral skeleton. J. Struct. Biol..

[34] Fischer-Cripps AC (2011). Nanoindentation.

[35] Deng X (2021). Topographically guided hierarchical mineralization. Mater. Today Bio.

[36] Oliver WC, Pharr GM, Introduction I (1992). An improved technique for determining hardness and elastic modulus using load and displacement sensing indentation experiments. J. Mater. Res..

[37] Nikolov S (2010). Revealing the design principles of high-performance biological composites using Ab initio and multiscale simulations: The example of lobster cuticle. Adv. Mater..

[38] Nečas D, Klapetek P (2012). Gwyddion: An open-source software for SPM data analysis. Cent. Eur. J. Phys..

[39] Greco G, Pugno NM (2020). Mechanical properties and weibull scaling laws of unknown spider silks. Molecules.

[40] Sawilowsky SS (2009). New effect size rules of thumb. J. Mod. Appl. Stat. Methods.

[41] Greco G, Wolff J, Pugno NM (2020). Strong and tough silk for resilient attachment discs: The mechanical properties of piriform silk, in the spider Cupiennius salei (Keyserling, 1877). Front. Mater..

[42] Xu M (2019). Easy, scalable, robust, micropatterned silk fibroin cell substrates. Adv. Mater. Interfaces.

[43] Neville AC (1975). Biology of the Arthropod Cuticle.

[44] Li C, Gorb SN, Rajabi H (2020). Cuticle sclerotization determines the difference between the elastic moduli of locust tibiae. Acta Biomater..

[45] Schmitt M, Büscher TH, Gorb SN, Rajabi H (2018). How does a slender tibia resist buckling? Effect of material, structural and geometric characteristics on buckling behaviour of the hindleg tibia in stick insect postembryonic development. J. Exp. Biol..

[46] Hepburn HR, Joffe I (1974). Locust solid cuticle-A time sequence of mechanical properties. J. Insect Physiol..

[47] Teyssié F (2015). Tarantulas of the world: Theraphosidae.

[48] Zuber M, Zia KM, Barikani M, Sabu T, Visakh PM, Aij PM (2013). Chitin and chitosan based blends, composites and nanocomposites. Advanced in Natural Polymers.

[49] Asgari M, Alderete NA, Lin Z, Benavides R, Espinosa HD (2021). A matter of size? Material, structural and mechanical strategies for size adaptation in the elytra of Cetoniinae beetles. Acta Biomater..

[50] Brekhovskikh LM, Goncharov V (1994). Mechanics of Continua and Wave Dynamics.

[51] Hawkins AD, Hazelwood RA, Popper AN, Macey PC (2021). Substrate vibrations and their potential effects upon fishes and invertebrates. J. Acoust. Soc. Am..

[52] Katritsis D (2007). Wall shear stress: Theoretical considerations and methods of measurement. Prog. Cardiovasc. Dis..

[53] Cheng L, Thomas A, Glancey JL, Karlsson AM (2011). Mechanical behavior of bio-inspired laminated composites. Compos. Part A Appl. Sci. Manuf..

[54] Das R (2022). The biomechanics of the locust ovipositor valves: A unique digging apparatus. J. R. Soc. Interface.

[55] Klocke D, Schmitz H (2011). Water as a major modulator of the mechanical properties of insect cuticle. Acta Biomater..

[56] Oyen ML, Shean TAVS, Strange DGT, Galli M (2012). Size effects in indentation of hydrated biological tissues. J. Mater. Res..

[57] Ebenstein DM (2011). Nano-JKR force curve method overcomes challenges of surface detection and adhesion for nanoindentation of a compliant polymer in air and water. J. Mater. Res.

[58] Chen SH, Soh AK (2008). The capillary force in micro- and nanoindentation with different indenter shapes. Int. J. Solids Struct..

[59] Oyen ML (2013). Nanoindentation of biological and biomimetic materials. Exp. Tech..

[60] Lakes R (2009). Viscoelastic Materials.

[61] Aberle B, Jemmali R, Dirks JH (2017). Effect of sample treatment on biomechanical properties of insect cuticle. Arthropod Struct. Dev..

[62] Gokoglu N, Topuz OK, Yerlikaya P, Yatmaz HA, Ucak I (2018). Effects of freezing and frozen storage on protein functionality and texture of some cephalopod muscles. J. Aquat. Food Prod. Technol..

