# Supplementary material for: The mechanical characterization of the legs, fangs, and prosoma in the spider Harpactira curvipes (Pocock 1897)
Source: Sci Rep. 2022 Jul 29;12:13056. doi: 10.1038/s41598-022-16307-y (PMC9338270; doi:10.1038/s41598-022-16307-y)
Supplement: Supplementary file 3 — Supplementary Information 3. [file 41598_2022_16307_MOESM3_ESM.docx]

**Supporting information**

**The mechanical characterization of the legs, fangs, and prosoma in the spider *Harpactira curvipes* (Pocock 1897)**

Sara Residori^1&^, Gabriele Greco^1&^ and Nicola M. Pugno^1,2^*

^1^ Laboratory for Bioinspired, Bionic, Nano, Meta Materials & Mechanics, Department of Civil, Environmental and Mechanical Engineering, University of Trento, Via Mesiano, 77, 38123 Trento, Italy

^2^ School of Engineering and Material Science, Queen Mary University of London, Mile End Road, E1 4NS London, United Kingdom

& These authors contributed equally

*corresponding author: [nicola.pugno@unitn.it](mailto:nicola.pugno@unitn.it)

**Table S1.** Values of the Young’s modulus and hardness at different spacing for the longitudinal basal section of the fang, and the epoxy resin used to prepare the samples.

| Spacing (μm) | Number of indentations | | Young’s modulus (GPa) | | p-value respect to 20 μm | | Hardness (GPa) | | p-value respect to 20 μm | |
| --- | --- | --- | --- | --- | --- | --- | --- | --- | --- | --- |
|  | Fang | Resin | Fang | Resin | Fang | Resin | Fang | Resin | Fang | Resin |
| 10 | 14 | 18 | 4.2±0.8 | 3.6±0.4 | 0.1573 | 0.1452 | 0.35±0.05 | 0.20±0.1 | 0.1262 | 0.2564 |
| 20 (used here) | 30 | 18 | 5.9±1.2 | 4.1±0.4 | / | / | 0.38±0.11 | 0.23±0.02 | / | / |
| 30 | 19 | 18 | 4.2±2.2 | 4.4±0.3 | 0.1601 | 0.2563 | 0.31±0.2 | 0.24±0.03 | 0.0960 | 0.6543 |
| 40 | 15 | 18 | 5.4±0.7 | 4.0±0.4 | 0.0815 | 0.3581 | 0.36±0.08 | 0.22±0.03 | 0.1002 | 0.8021 |

**Table S2.** Values of the Young’s modulus and hardness for different sections of the prosoma.

|  | Outer layer |
| --- | --- |
| Number of indentations | 31 |
| Avg. Young’s modulus [GPa] | 3.42±0.57 |
| Avg. Hardness [GPa] | 0.34±0.07 |
| Avg. Depth [nm] | 3042±242 |

**Table S3.** Values of the Young’s modulus and hardness for different sections of legs.

|  | Outer layer |
| --- | --- |
| Number of indentations | 173 |
| Avg. Young’s modulus [GPa] | 7.20±1.18 |
| Avg. Hardness [GPa] | 0.32±0.09 |
| Avg. Depth [nm] | 2663±403 |

**Table S4.** Values of the Young’s modulus and hardness for different sections of the fang in the longitudinal direction.

| LONGITUDINAL DIRECTION | | | | | | |
| --- | --- | --- | --- | --- | --- | --- |
|  | TIP SECTION | | CROSS SECTION | | BASIS SECTION | |
|  | Inner layer | Outer layer | Inner layer | Outer layer | Inner layer | Outer layer |
| Number of indentations | 175 | 42 | 87 | 35 | 107 | 28 |
| Avg. Young’s modulus [GPa] | 5.60±0.36 | 6.20±0.52 | 4.02±0.15 | 4.11±0.35 | 3.64±0.27 | 3.60±0.24 |
| Avg. Hardness [GPa] | 0.53±0.07 | 0.68±0.05 | 0.44±0.03 | 0.44±0.06 | 0.40±0.04 | 0.39±0.05 |
| Avg. Depth [nm] | 2209±135 | 1996±172 | 2466±170 | 2514±164 | 2569±254 | 2666±398 |

**Table S5.** Values of the Young’s modulus and hardness for different sections of the fang in the transversal direction.

| TRANSVERSAL DIRECTION | | | | |
| --- | --- | --- | --- | --- |
|  | TIP SECTION | | BASIS SECTION | |
|  | Inner layer | Outer layer | Inner layer | Outer layer |
| Number of indentations | 187 | 56 | 165 | 166 |
| Avg. Young’s modulus [GPa] | 6.57±0.59 | 7.56±0.48 | 3.66±0.36 | 3.15±0.69 |
| Avg. Hardness [GPa] | 0.49±0.05 | 0.59±0.08 | 0.22±0.02 | 0.21±0.03 |
| Avg. Depth [nm] | 2437±58 | 2216±43 | 2334±559 | 2463±178 |

**Table S6-7 are reported below in the end of the file.**

**Table S8.** Roughness values.

| Ra [nm] | Rq [nm] |
| --- | --- |
| 6.7 ± 0.9 | 8.5 ± 1.1 |


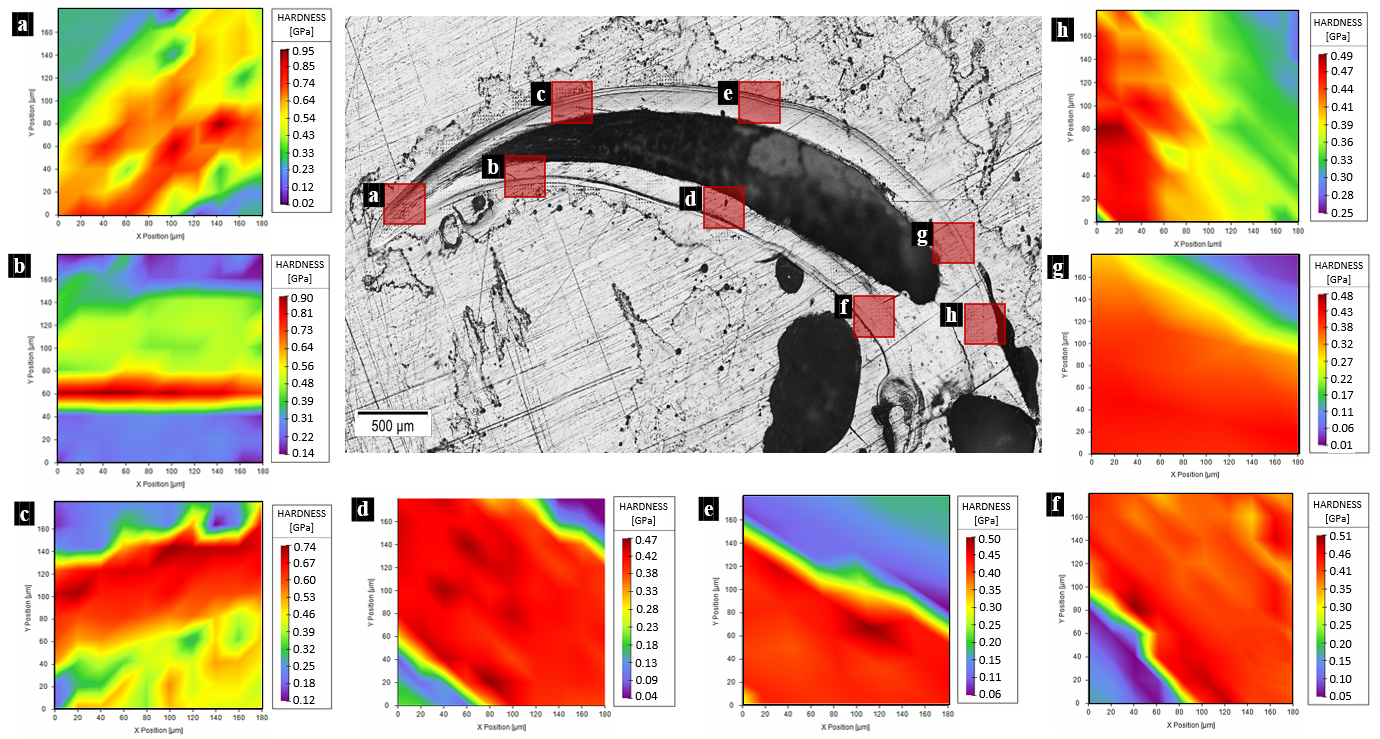


**Figure S1.** Map of hardness in different sections of the spider fang in the longitudinal direction. Images generated with the support of Nanoblitz 3d, Nanomechanics Inc.


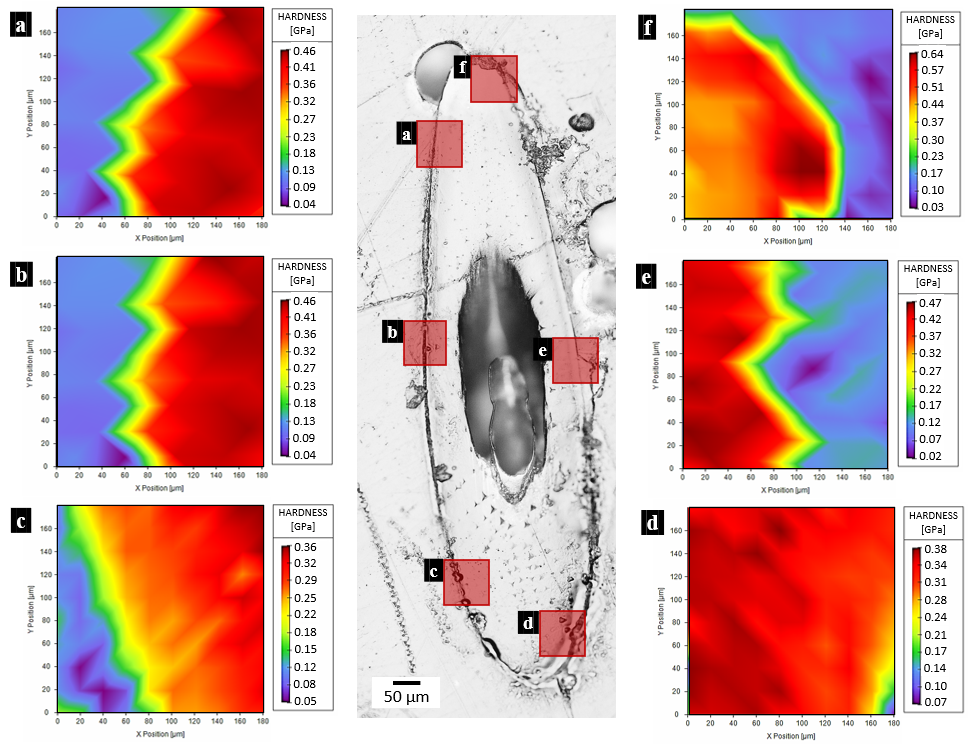


**Figure S2.** Map of hardness of the spider fang in the transversal section on the tip. Images generated with the support of Nanoblitz 3d, Nanomechanics Inc.


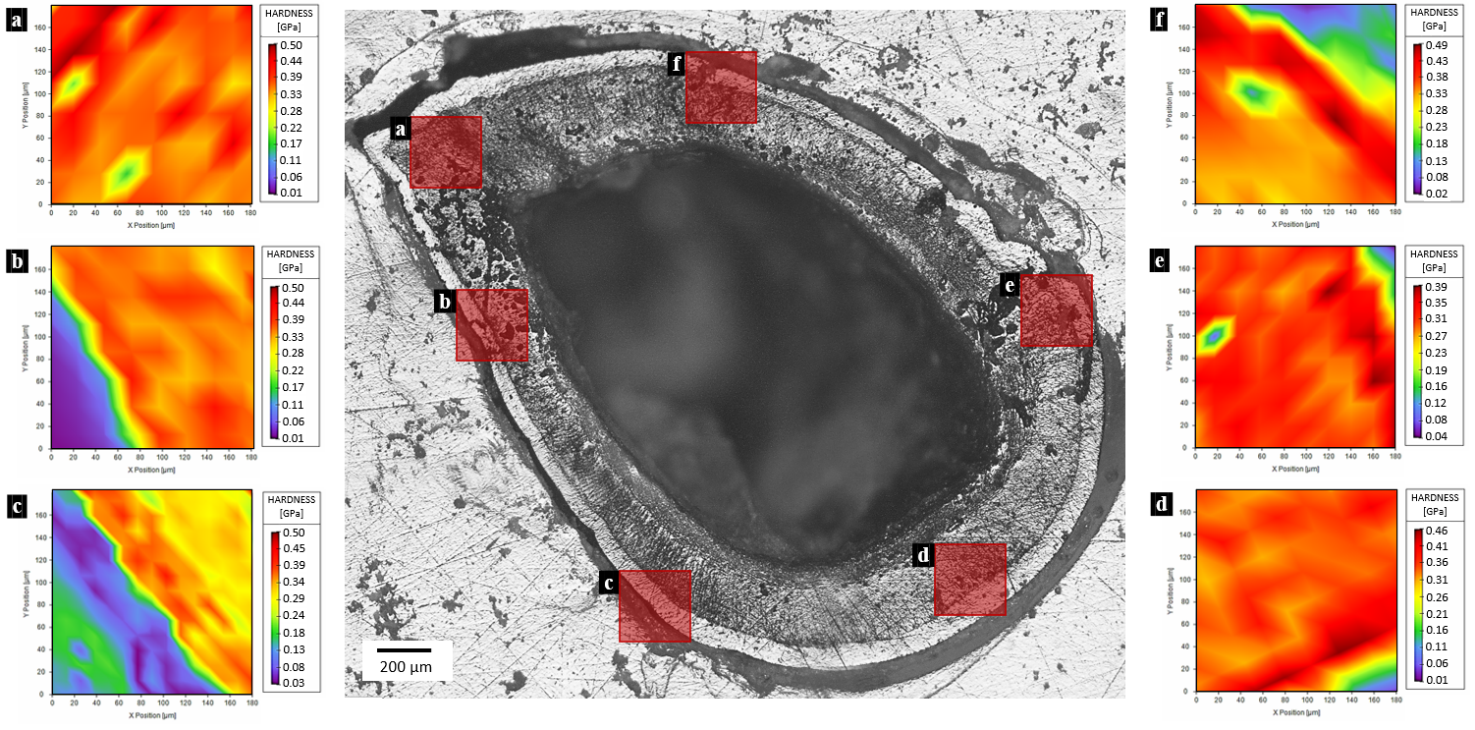


**Figure S3.** Map of hardness of the spider fang in the transversal section on the base. Images generated with the support of Nanoblitz 3d, Nanomechanics Inc.


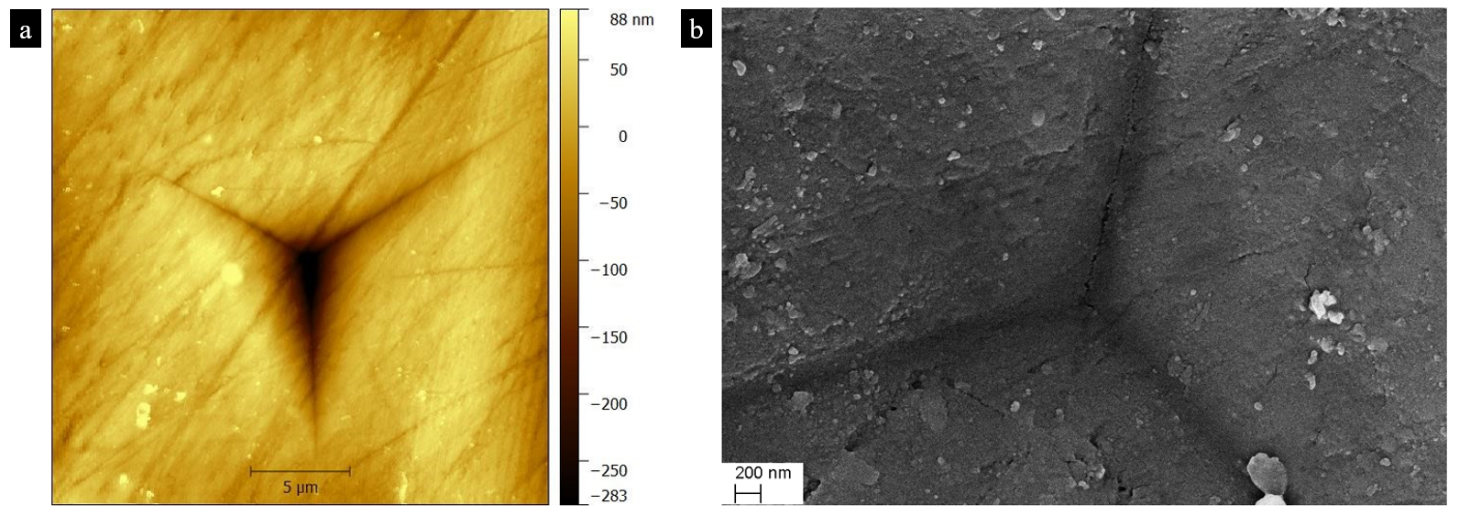


**Figure S4.** Images of the indentation print obtained with a) AFM and b) SEM.


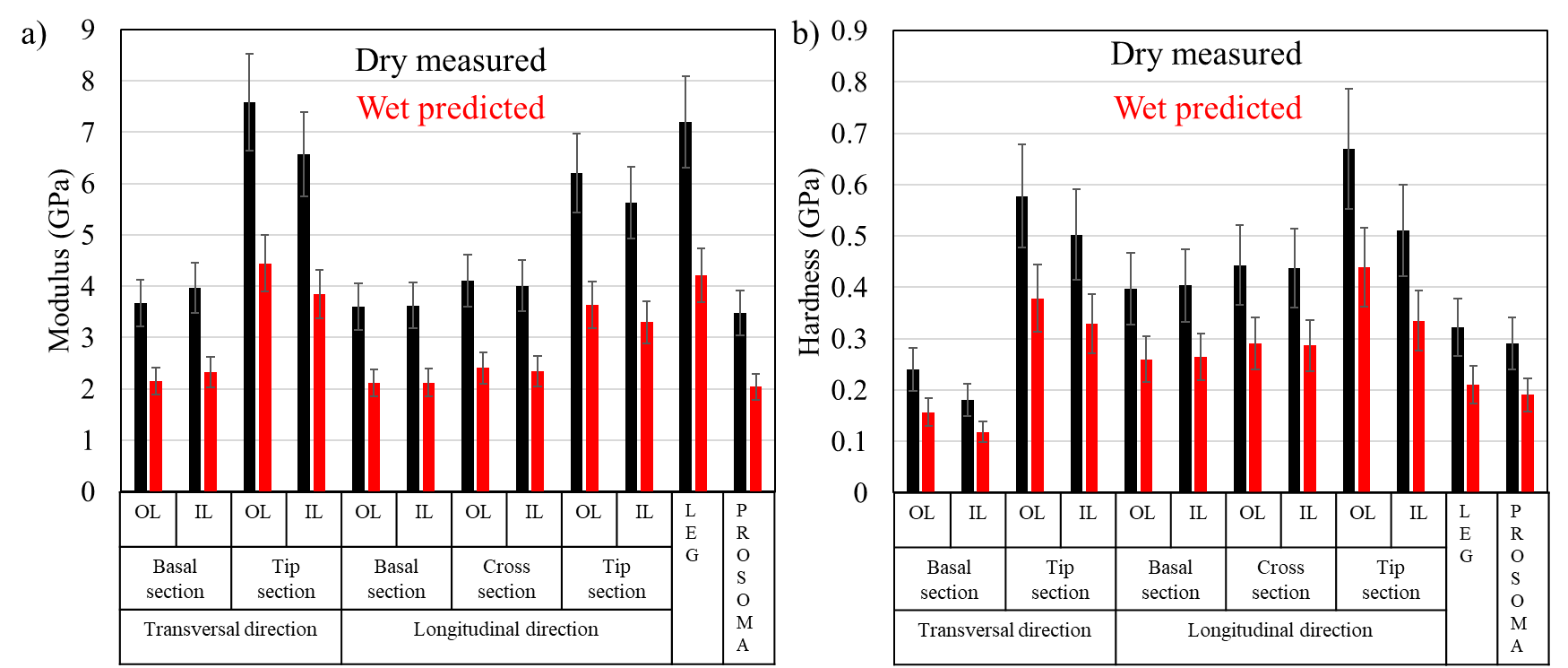


**Figure S5.** Avearges values of the a) Young’s modulus and b) the hardness obtained in this study with nanoindentation and those in wet state predicted using the values in Tadayon et al.^19^. In this case, we averaged the mean relative differences and used them to estimate the reduction in the mechanical properties due to hydration. In particular, for hardness a reduction of 35% has been used and for Young’s modulus a reduction of 41.4%.

Table S6: p-values and Cohen coefficient (in italic) of the comparison of Young’s modulus among the different body parts of the spider.

| Young’s modulus | | | TRANSVERSAL DIRECTION | | | | LONGITUDINAL DIRECTION | | | | | | LEG | PROSOMA |
| --- | --- | --- | --- | --- | --- | --- | --- | --- | --- | --- | --- | --- | --- | --- |
|  |  |  | BASIC SECTION | | TIP SECTION | | BASIC SECTION | | CROSS SECTION | | TIP SECTION | |  |  |
|  |  |  | Outer layer | Inner layer | Outer layer | Inner layer | Outer layer | Inner layer | Outer layer | Inner layer | Outer layer | Inner layer | Outer layer | Outer layer |
| TRANSVERSAL DIRECTION | BASIC SECTION | Outer layer | - | 0.0030  *0.3570* | 0.0000 *5.2191* | 0.0000 *3.8100* | 0.6530  *0.0944* | 0.5839  *0.0727* | 0.0006  *0.6196* | 0.0002  *0.5315* | 0.0000 *3.2477* | 0.0000 *2.5375* | 0.0000  *3.1756* | 0.2373  *0.2487* |
|  |  | Inner layer |  | - | 0.0000  *4.3784* | 0.000 *3.1676* | 0.0284*0.4513* | 0.0001  *0.4941* | 0.3285  *0.1677* | 0.7240  *0.0468* | 0.0000  *2.6234* | 0.0000  *2.0291* | 0.0000  *2.9147* | 0.0048  *0.5838* |
|  | TIP SECTION | Outer layer |  |  | - | 0.0000  *1.4769* | 0.0000  *7.3601* | 0.0000  *9.6443* | 0.0000 *6.7807* | 0.0000  *8.0571* | 0.0000 *2.0305* | 0.0000 *2.6755* | 0.0329  *0.3300* | 0.0000 *6.8505* |
|  |  | Inner layer |  |  |  | - | 0.0000*4.5535* | 0.0000  *5.5430* | 0.0000  *3.9328* | 0.0000*4.5685* | 0.0071  *0.4966* | 0.0000  *1.2588* | 0.0000  *0.5736* | 0.0000  *4.5265* |
| LONGITUDINAL DIRECTION | BASIC SECTION | Outer layer |  |  |  |  | - | 0.6086  *0.1090* | 0.0000  *1.7746* | 0.0000  *1.5368* | 0.0000 *4.3849* | 0.0000 *2.8564* | 0.0000 *2.9964* | 0.2927  *0.2840* |
|  |  | Inner layer |  |  |  |  |  | - | 0.0000 *1.9795* | 0.0000 *1.5906* | 0.0000 *6.0094* | 0.0000  *3.2778* | 0.0000  *3.4979* | 0.0288  *0.4691* |
|  | CROSS SECTION | Outer layer |  |  |  |  |  |  | - | 0.0509  *0.3674* | 0.0000  *3.7900* | 0.0000 *2.2093* | 0.0000 *2.6647* | 0.0000  *1.5640* |
|  |  | Inner layer |  |  |  |  |  |  |  | - | 0.0000  *4.7047* | 0.0000 *2.5528* | 0.0000 *3.0079* | 0.0000  *1.5309* |
|  | TIP SECTION | Outer layer |  |  |  |  |  |  |  |  | - | 0.0000  *0.7651* | 0.0000 *0.8253* | 0.0000 *4.1444* |
|  |  | Inner layer |  |  |  |  |  |  |  |  |  | - | 0.0000  *1.4867* | 0.0000 *2.9468* |
| LEG | | Outer layer |  |  |  |  |  |  |  |  |  |  | - | 0.0000 *3.0667* |
| PROSOMA | | Outer layer |  |  |  |  |  |  |  |  |  |  |  | - |

Table S7: p-values and Cohen coefficient (in italic) of the comparison of hardness among the different body parts of the spider.

| Hardness | | | TRANSVERSAL DIRECTION | | | | LONGITUDINAL DIRECTION | | | | | | LEG | PROSOMA |
| --- | --- | --- | --- | --- | --- | --- | --- | --- | --- | --- | --- | --- | --- | --- |
|  |  |  | BASIC SECTION | | TIP SECTION | | BASIC SECTION | | CROSS SECTION | | TIP SECTION | |  |  |
|  |  |  | Outer layer | Inner layer | Outer layer | Inner layer | Outer layer | Inner layer | Outer layer | Inner layer | Outer layer | Inner layer | Outer layer | Outer  layer |
| TRANSVERSAL DIRECTION | BASIC SECTION | Outer layer | - | 0.0000  *0.8716* | 0.0000 *4.7272* | 0.0000 *3.5767* | 0.0000  *2.2337* | 0.0000  *2.8274* | 0.0000  *3.0462* | 0.0000  *3.3127* | 0.000 *5.3582* | 0.0000 *3.8241* | 0.0000*0.8754* | 0.0028  *0.6370* |
|  |  | Inner layer |  | - | 0.0000 *6.4694* | 0.0000  *4.9290* | 0.0000  *3.6843* | 0.0000  *4.4160* | 0.0000*4.6679* | 0.0000  *4.9859* | 0.0000 *7.1149* | 0.0000 *5.1148* | 0.0000  *1.6370* | 0.0000 *1.6333* |
|  | TIP SECTION | Outer layer |  |  | - | 0.0000  *1.1119* | 0.0000  *3.2932* | 0.0000 *4.1840* | 0.0000  *2.7176* | 0.0000  *3.3026* | 0.0000  *1.2081* | 0.0000  *1.0137* | 0.0000 *2.6705* | 0.0000 *3.8658* |
|  |  | Inner layer |  |  |  | - | 0.0000  *1.6073* | 0.0000 *1.8457* | 0.0000  *0.9637* | 0.0000  *1.1767* | 0.0000  *2.1619* | 0.3160  *0.1228* | 0.0000 *1.9414* | 0.0000 *2.7732* |
| LONGITUDINAL DIRECTION | BASIC SECTION | Outer layer |  |  |  |  | - | 0.2672  *0.2365* | 0.0000  *1.4204* | 0.0000  *1.4725* | 0.0000 *3.5980* | 0.0000 *1.7716* | 0.0002  *0.7656* | 0.0000  *1.4629* |
|  |  | Inner layer |  |  |  |  |  | - | 0.0000  *1.4793* | 0.0000  *1.3698* | 0.0000 *4.9636* | 0.0000 *1.9283* | 0.0000  *0.9794* | 0.0000  *2.3205* |
|  | CROSS SECTION | Outer layer |  |  |  |  |  |  | - | 0.2666  *0.2080* | 0.0000  *3.3414* | 0.0000 *1.1013* | 0.0000  *1.2817* | 0.0000 *2.4018* |
|  |  | Inner layer |  |  |  |  |  |  |  | - | 0.0000  *4.1491* | 0.0000 *1.2880* | 0.0000  *1.3424* | 0.0000  *2.8951* |
|  | TIP SECTION | Outer layer |  |  |  |  |  |  |  |  | - | 0.0000 *2.1832* | 0.0000 *3.4021* | 0.0000 *4.0494* |
|  |  | Inner layer |  |  |  |  |  |  |  |  |  | - | 0.0000 *2.1541* | 0.0000 *3.0703* |
| LEG | | Outer layer |  |  |  |  |  |  |  |  |  |  | - | 0.1389  *0.3027* |
| PROSOMA | | Outer layer |  |  |  |  |  |  |  |  |  |  |  | - |
